# Optimization of the Boundary Conditions of a Board Level Reliability Test Board to Maximize the Fatigue Life of Ball Grid Array Solder Joints under Thermal Cycling and Random Vibration

**DOI:** 10.3390/ma17030755

**Published:** 2024-02-04

**Authors:** Jisup Lee, Hyunsik Jeong, Gunhee Jang

**Affiliations:** Department of Mechanical Convergence Engineering, Hanyang University, Seoul 04763, Republic of Korea; jisup0813@hanyang.ac.kr (J.L.); plusboy111@hanyang.ac.kr (H.J.)

**Keywords:** boundary condition, fatigue life, multi-objective optimization, solder joint

## Abstract

We investigated the screw hole position of a board level reliability (BLR) test board to improve the fatigue reliability of solder joints under thermal cycling and random vibration. We developed a finite element model of a BLR test board and derived the plastic strain energy density and 1-sigma stress, which are the main parameters influencing the fatigue life of solder joints under thermal cycling and random vibration, respectively. We analyzed the correlation between the screw hole position and the main parameters of the fatigue life through sensitivity analysis. By performing multi-objective optimization, we determined the screw hole position that maximizes the fatigue life of solder joints under thermal cycling and random vibration. With the optimal screw hole position, the fatigue life significantly increased under thermal cycling and random vibration compared to the BLR test board with the initial screw hole position.

## 1. Introduction

Ball grid array (BGA) packages are exposed to various external environments during manufacturing, transport, and operation. Solder balls, which are components of BGA packages, can be damaged easily when exposed to continuous and repetitive loads. Electronic packaging failure has been attributed to temperature (55%), vibration (20%), humidity (19%), and dust (6%); electronic packaging is most vulnerable to temperature cycling and vibration conditions [1]. Many researchers have conducted simulations and experiments to evaluate and predict the fatigue life of solder joints under temperature and vibration as a measure of board level reliability (BLR) [2,3,4,5,6,7]. Their efforts have made it possible to predict the fatigue life of solder joints accurately and ensure the robustness and durability of solder balls. Chen et al. [2] calculated the stress on a solder ball using finite element analysis (FEA) because it is difficult to measure the exact stress on a small-sized solder ball in a vibration experiment. The S-N curve was obtained based on calculated stress and vibration experiments, and the damage to the solder joint was calculated by using the S-N curve. Jang et al. [3] predicted the fatigue life of a dummy solder ball for a solid-state drive (SSD) under vibration loading. Through FEA and vibration experiments, the S-N curve of the dummy solder ball was derived, and the solder ball at the corner of the package was found to be the most vulnerable. Xia et al. [4] derived the S-N curve for solder joints in a package-on-package (PoP) assembly using FEA and sine-sweep experiments. In addition, the fatigue life of solder joints was calculated by using Palmgren–Miner’s rule under random vibration, and the calculation results were verified via random vibration experiments. Fatigue failure of solder joints under thermal cycling has been identified as a cause of a coefficient of thermal expansion (CTE) mismatch between the chip and PCB substrate. Syed et al. [5] derived the material constants of solder joints for the life prediction model by curve-fitting the creep strain calculated from simulations and the mean cycles to failure from actual test. Based on this model, Depiver et al. [6] calculated the fatigue life of Sn-Pb and Sn-Ag-Cu solder joints under thermal cycling. Their study showed that the fatigue life of Sn-Pb solder joints was short because the strain energy of Sn-Pb solder joints was higher than that of Sn-Ag-Cu solder joints. Chen et al. [7] analyzed the fatigue of individual solder joints under rapid thermal cycling. Thermal deformation occurs due to rapid temperature change in the solder joint, and cracks on the surface of the solder joint propagate where the stress concentrations caused by surface oxidation, intermetallic compound (IMC), and CTE mismatch of between the solder and the pad are the greatest.

Recently, many studies have been conducted on the changing mechanical design of PCB and the boundary conditions that improve the fatigue life of solder joints [8,9,10]. Doranga et al. [8] compared the fatigue life of solder joints with different PCB thicknesses under vibration loading. They found that the natural frequency and stiffness of the board increase as the thickness of the board increases. As a result, the stress on the solder joint decreases and the fatigue life increases. Jeong et al. [9] investigated the effect of the fastening area of a board with mounted packages on the fatigue life of solder joints under random vibration. They found that the stiffness of the board increases as the fastening area increases, resulting in increased solder joint lifetime. Wenchao et al. [10] investigated the change in the fatigue life of solder joints due to the locations of screw holes used to fix the PCB board under thermal cycling. The plastic strain of the solder joint for five different screw hole locations was calculated through simulations. It was confirmed that the plastic strain decreases when the screw hole locations are closer to the package, increasing the fatigue life. In addition, the simulation results were verified through experiments. Many studies have investigated the relationship between the fatigue life of solder joints and boundary conditions under either vibration or thermal cycling environments, but there is a lack of research analyzing the boundary conditions that improve the fatigue life of solder joints subjected to both environments.

To improve the reliability of the BGA solder joints mounted on a BLR test board under thermal cycling and random vibration, we optimized the screw hole position, which is a boundary condition for BLR test boards. We developed a finite element model of the BLR test board and derived the plastic strain energy density and the 1-sigma stress, which are the main parameters of the fatigue life of solder joints under thermal cycling and random vibration, respectively. In addition, we analyzed the correlation between the screw hole position and the main parameters through sensitivity analysis and developed an approximate model to optimize the screw hole position. Thus, the fatigue life of solder joints was maximized under thermal cycling and random vibration, and a method to improve the solder joint fatigue life under both environments was developed.

## 2. Finite Element Analysis

### 2.1. Fatigue Life Model for Solder Joints

Repetitive loads, such as thermal cycling and vibration, are the main factors causing the fatigue failure of solder joints. By studying the relationship between stress and the number of cycles to failure, the fatigue life of solder joints can be predicted. Solder joint failure under thermal cycling results from low-cycle fatigue (1–10^4^ cycles to failure). The Morrow energy-based fatigue model is most widely used to predict the lifetime of low-cycle fatigue, as shown in Equation (1) [11]:

(1) $${\left(N_{f,TC}\right)}^{m_{f}}\times \Delta W_{p}=K$$

where $N_{f,TC}$ is the number of cycles to failure, $m_{f}$ is the fatigue exponent, and K is the fatigue ductility coefficient. $\Delta W_{p}$ is the accumulated plastic strain energy density (PSED) per cycle, calculated through FEA.

Solder joint failure under vibration results from high-cycle fatigue (10^3^–10^8^ cycles to failure). The Basquin equation represents the relationship between stress range and the number of cycles to failure due to vibration and is used to predict high-cycle fatigue. The Basquin equation is shown in Equation (2) [12]:

(2) $$\sigma_{a}=\sigma_{f}^{\prime }{\left(2N_{f,vib.}\right)}^{b}$$

where $\sigma_{a}$ is the amplitude of stress, $\sigma_{f}^{\prime }$ is the fatigue strength coefficient, $2N_{f,vib.}$ is the number of reversals to failure, and b is the fatigue strength exponent. In addition, Steinberg three-band technology is used to predict solder joint fatigue life under random vibration. Steinberg [13] developed an empirical model to determine the component life under vibration. The model assumes that the vibration load follows a Gaussian distribution and that the response stress or strain of 1-sigma, 2-sigma, and 3-sigma occurs at 68.31%, 27.1%, and 4.33% of the time, respectively. The Steinberg three-band equation combined with the Basquin equation is as follows:

(3) $$T_{failure}=\frac{\frac{1}{2}{\left(\sigma_{f}^{\prime }\right)}^{-1/b}}{f_{1}\times {\left(\sigma_{1\mathrm{sigma}}\right)}^{-1/b}\times \left[0.683+0.271\times 2^{-1/b}+0.043\times 3^{-1/b}\right]}$$

where $T_{failure}$ is the time to failure and $f_{1}$ is the first natural frequency. $\sigma_{1\mathrm{sigma}}$ is the 1-sigma stress and is calculated through FEA.

This study used the Morrow energy-based fatigue model and the Steinberg three-band equation to predict the fatigue life of solder joints under thermal cycling and random vibration, respectively. According to these models, the fatigue life of solder joints can be increased by reducing the accumulated PSED per cycle and 1-sigma stress. Based on these results, we set the accumulated PSED per cycle and 1-sigma stress as the main fatigue life parameters and investigated the relationship between the screw hole position of the BLR test board and the main fatigue life parameters.

### 2.2. Finite Element Model

**Figure 1:**
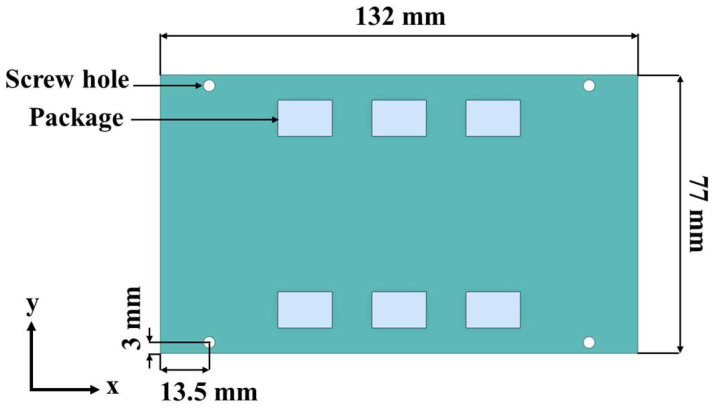
Layout of the BLR test board.

**Figure 2:**
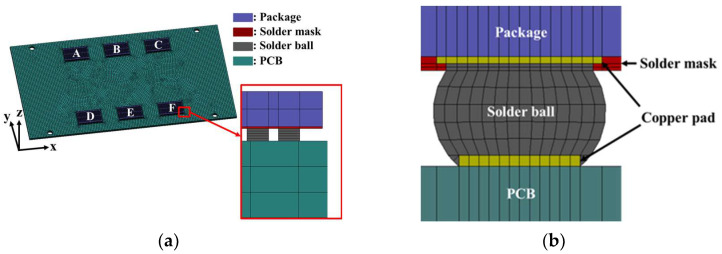
Finite element model geometry: (**a**) BLR test board; (**b**) detailed cross-section of solder joint.

The BLR test board used in this study is shown in Figure 1. The board size followed the Joint Electron Device Engineering Council (JEDEC) standard, JESD22-B111 [14]. The width and length of the board were 77 mm and 132 mm, respectively, and the thickness was 1.6 mm. The screw holes were located at 13.5 mm in the x-direction and 3 mm in the y-direction from the corner of the PCB. Each package was connected to the PCB with 200 BGA solder balls. Figure 2a shows the finite element model of the BLR test board developed using the commercial software ANSYS 2022 R1. The model consisted of six packages, solder masks, solder balls, and the PCB. The packages are represented as A to F. 

To reduce the simulation time, all solder balls were modeled as equivalent cubes. The total number of elements used in the finite element model was 103,797. Simulations of thermal cycling and random vibration were performed using the developed finite element model to identify the location of the most vulnerable solder joint and determine the response of the solder joint. In addition, the detailed structure of the solder joint shown in Figure 2b was reflected in the vulnerable solder joint to accurately predict the fatigue life. Fatigue life prediction using the detailed solder joint is explained in Section 3. The solder ball material was SAC305 (Sn-3.0Ag-0.5Cu wt.%). All materials were assumed to exhibit elastic behavior under vibration loading. However, when considering thermal loading, solder balls were assumed to follow viscoplastic behavior due to their low melting point. The Anand model, proposed by Anand [15] and Brown [16], was used to represent the viscoplastic behavior of the solder ball. Initially, the model was developed for high-temperature metal-forming methods, such as deep-drawing and rolling. It has been extended to predict the lifetime of solder joints in electronic packaging [6]. The Anand model is represented by the flow equation and the evolution equation as follows:

(4) $${\dot{\epsilon }}_{p}=Ae^{-\frac{Q}{RT}}{\left[\sin h\left(\xi \frac{\sigma }{s}\right)\right]}^{\frac{1}{m}}$$

(5) $$\dot{s}=\left\{h_{0}{\left|1-\frac{s}{s^{*}}\right|}^{a}\mathrm{sign}\left(1-\frac{s}{s^{*}}\right)\right\}{\dot{\epsilon }}_{p}$$

where

(6) $$s^{*}=\hat{s}{\left(\frac{{\dot{\epsilon }}_{p}}{A}e^{\frac{Q}{RT}}\right)}^{n}$$

where ${\dot{\epsilon }}_{p}$ is the inelastic strain rate, σ is the effective true stress, A is the pre-exponential factor, Q is the activation energy, m is the strain rate sensitivity, ξ is the multiplier of stress, R is the universal gas constant, T is the absolute temperature, s is the initial value of deformation resistance, $h_{0}$ is the hardening constant, $\hat{s}$ is the coefficient for the deformation resistance saturation value, n is the strain rate sensitivity of saturation value, and a is the strain rate sensitivity of hardening. Table 1 shows the material properties of the BLR test board [3,4,17,18,19,20]. The Anand model constants for SAC305 [21] are shown in Table 2.

### 2.3. Thermal Cycling Analysis

**Figure 3:**
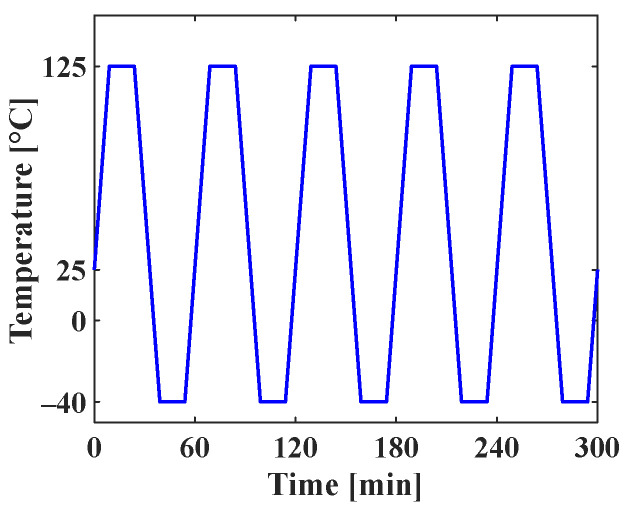
Applied thermal cycling profile.

**Figure 4:**
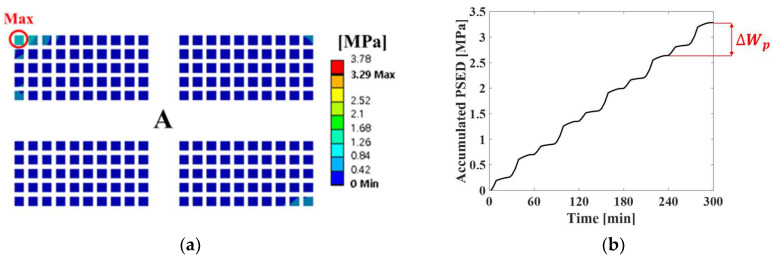
Results of thermal cycling analysis: (**a**) contour plot of the accumulated PSED after five cycles and location of the maximum accumulated PSED on the A package; (**b**) time series of accumulated PSED of solder joint at the location where the maximum accumulated PSED occurred.

Thermal cycling analysis was performed by using the finite element model to calculate the PSED of the solder joint. Figure 3 shows the applied thermal cycling profile of G conditions in JESD22-A104F.01 [22]. The room temperature was 25 °C, and the minimum and maximum temperatures were −40 °C and 125 °C, respectively. In addition, the ramp rate was 11 °C/min, and dwell time was 15 min. Thermal cycling was simulated for five cycles. The displacements in the x-, y-, and z-directions for the four screw holes were fixed as boundary conditions. Figure 4a shows the contour plot of the accumulated PSED and location of the maximum accumulated PSED after five cycles. The solder joint at the outermost corner of the A package had the greatest maximum accumulated PSED, and it was located nearest to the screw hole. These results are consistent with the results of Oh et al. [23]. Figure 4b shows a time series of the accumulated PSED of the solder joint where the maximum accumulated PSED appeared. The accumulated PSED per cycle of the solder joint was calculated as 0.65 MPa.

### 2.4. Random Vibration Analysis

**Figure 5:**
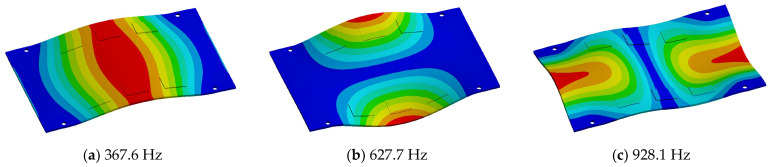
Natural frequencies and mode shapes from FEA: (**a**) 1st mode; (**b**) 2nd mode; (**c**) 3rd mode.

**Figure 6:**
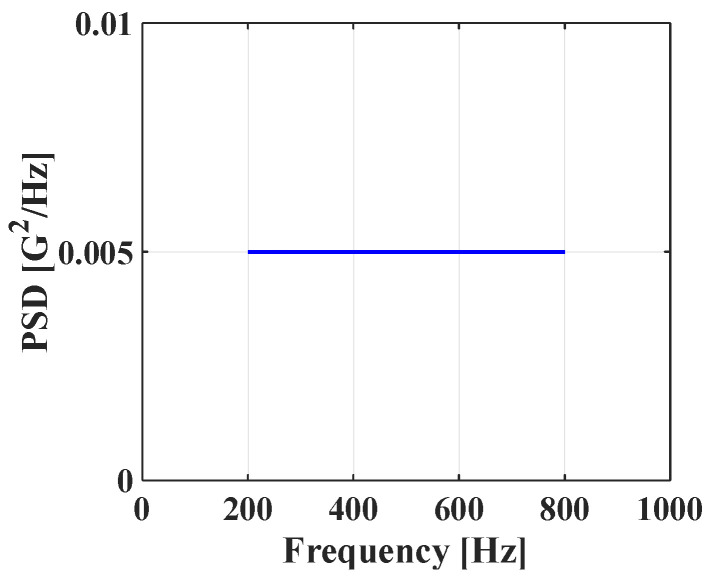
Applied PSD profile.

**Figure 7:**
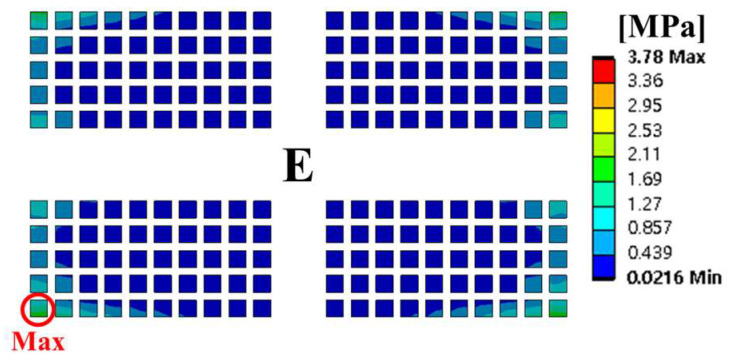
Contour plot of 1-sigma stress and location of the maximum 1-sigma stress on E package under random vibration.

Random vibration analysis was performed to calculate the 1-sigma von Mises stress of the solder joint. First, modal analysis was conducted to determine the natural frequencies of the BLR test board before performing the random vibration analysis. Four screw holes were fixed as boundary conditions. Figure 5 shows the natural frequencies and mode shapes. The damping ratio of the BLR test board was set to 2%, which was derived from the measured frequency response function through modal experiments by Jeong et al. [9]. Figure 6 shows the applied power spectral density (PSD) profile. PSD acceleration was applied to the screw holes in the z-direction. Considering that the natural frequency may change depending on the screw hole position, the PSD was maintained at a constant level of 1.73 G_rms_ within the frequency range of 200–800 Hz. Figure 7 shows the contour plot of 1-sigma stress and location of the maximum 1-sigma stress for the E package. A maximum value of 3.78 MPa was calculated at the outermost corner solder joint of the E package, located in the center of the BLR test board. This result is consistent with the findings of An et al. [24]. Vibration excitation resulted in bending of the PCB, and the solder joint located in the center of the PCB was the most vulnerable due to the greatest curvature radius occurring at the center of the PCB.

## 3. Optimization of the Screw Hole Position

### 3.1. Sensitivity Analysis

**Figure 8:**
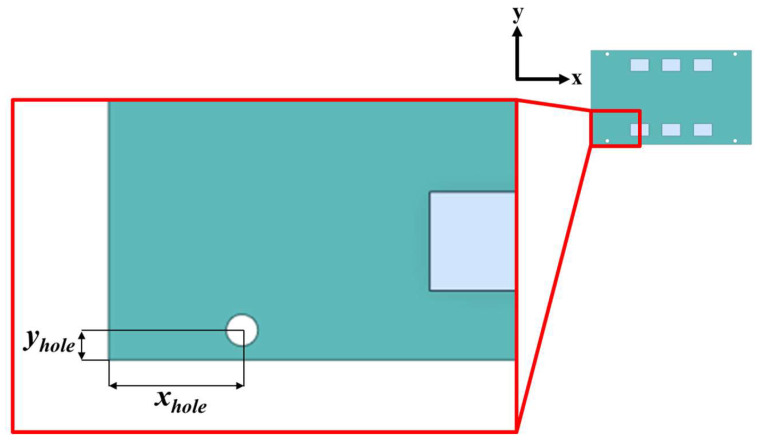
Design variables of the BLR test board.

**Figure 9:**
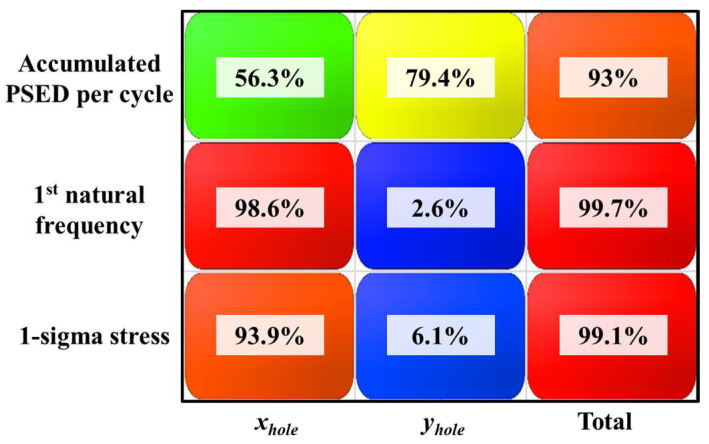
CoP matrix.

**Figure 10:**
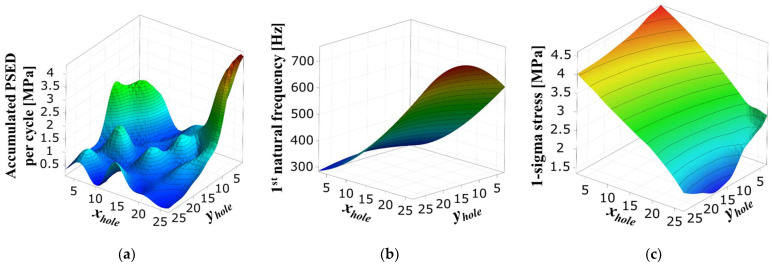
Three-dimensional plot of the response surface: (**a**) accumulated PSED per cycle; (**b**) 1st natural frequency; (**c**) 1-sigma stress.

A sensitivity analysis was performed to investigate the correlation between the screw hole position and the main parameters of the fatigue life, which are the accumulated PSED per cycle and 1-sigma stress. Figure 8 shows the design variables representing the distance of the screw hole from the PCB corner. The center coordinates of the four screw holes move symmetrically relative to the center of the BLR test board. Table 3 shows the upper and lower limits of the design variables. The output variables are the accumulated PSED per cycle, first natural frequency, and 1-sigma stress. As the first step of the sensitivity analysis, sampling points were generated by using the Latin hypercube sampling method. A total of 105 sampling points were used to perform thermal cycling, modal analysis, and random vibration analysis. In the second step, we created the metamodel of optimal prognosis (MOP) using commercial software (ANSYS optiSLang 2022 R1). The MOP creates an approximate model of the output variables for design variables that shows the correlation between the design and output variables. The prediction quality of an approximate model is expressed using the coefficient of prognosis (CoP). In the third step, a sensitivity analysis was performed by using the 3D response surface generated from the MOP. Figure 9 shows the CoP matrix. It can be observed that the accumulated PSED per cycle is greatly influenced by the design variables *x_hole_* and *y_hole_*, whereas the design variable for the 1-sigma stress and the first natural frequency is *x_hole_*. The last column displays the full model CoPs. The generated approximate model has a high prediction quality of over 93%. Figure 10 shows the 3D response surface and the correlation between design variables and output variables. In Figure 10a, there is a nonlinear relationship between design variables and the accumulated PSED per cycle, and increasing both *x_hole_* and *y_hole_* results in a decrease in the accumulated PSED per cycle. Figure 10b shows that the first natural frequency increases as *x_hole_* increases. Figure 10c shows that the 1-sigma stress decreases as *x_hole_* increases. The increase of natural frequency results from a higher board stiffness, consequently decreasing the 1-sigma stress.

### 3.2. Multi-Objective Optimization

**Figure 11:**
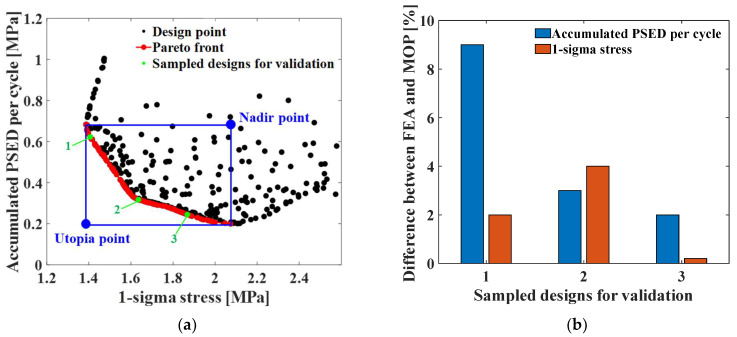
Optimization results: (**a**) plot of the Pareto front; (**b**) difference between FEA and MOP.

To maximize the fatigue life of solder joints, multi-objective optimization was performed to minimize the accumulated PSED per cycle and the 1-sigma stress. The formulation of the optimization problem is as follows:

$$\mathrm{Minimize}\;\Delta W_{p},\sigma_{1\mathrm{sigma}}$$

(7) $$\mathrm{Subject}\;\mathrm{to}\;x_{hole}^{lower}\leq x_{hole}\leq x_{hole}^{upper}\;\;\;\;\;\;\;\;\;\;\;\;\;\;\;\;\;\;\;\;\;y_{hole}^{lower}\leq y_{hole}\leq y_{hole}^{upper}$$

where $x_{hole}^{upper}$, $x_{hole}^{lower}$, $y_{hole}^{upper}$, and $y_{hole}^{lower}$ are the upper and lower limits of the design variables and are the same as the values used in the sensitivity analysis. $\Delta W_{p}$ and $\sigma_{1\mathrm{sigma}}$ are the accumulated PSED per cycle and the 1-sigma stress, respectively, which are objective functions. A multi-objective optimization problem was solved using the evolutionary algorithm (EA) based on the MOP generated from the sensitivity analysis. Figure 11a shows the Pareto front, which is the result of the multi-objective optimization. The two objective functions show a tradeoff relationship. To verify the prediction quality of the MOP and the Pareto front, three points were randomly selected from the Pareto front, and we compared the difference of the two objective functions calculated using FEA and MOP, as shown in Figure 11b. The differences in the accumulated PSED per cycle and 1-sigma stress between MOP and FEA were less than 10%, which confirmed the accuracy of MOP and the Pareto front. To select the final design from the Pareto front, we used the weighted sum method. The formulation is as follows:

$$\mathrm{Minimize}\;\alpha \frac{\Delta W_{p}-\Delta W_{p}^{U}}{\Delta W_{p}^{N}-\Delta W_{p}^{U}}+\left(1-\alpha \right)\frac{\sigma_{1\mathrm{sigma}}-{\sigma_{1\mathrm{sigma}}}^{U}}{{\sigma_{1\mathrm{sigma}}}^{N}-{\sigma_{1\mathrm{sigma}}}^{U}}$$

(8) $$\mathrm{Subject}\;\mathrm{to}\;x_{hole}^{lower}\leq x_{hole}\leq x_{hole}^{upper}\;\;\;\;\;\;\;\;\;\;\;\;\;\;\;\;\;\;\;\;\;y_{hole}^{lower}\leq y_{hole}\leq y_{hole}^{upper}\;\;\;\;\alpha \in \left[0,\;1\right]$$

where α is the weighting factor. The failure sources of electronic packaging are temperature (55%), vibration (20%), humidity (19%), and dust (6%) [1]. By considering the failure rates for temperature and vibration, the weighting factor was calculated as 0.73. $\Delta W_{p}^{U}$ and ${\sigma_{1\mathrm{sigma}}}^{U}$ are the accumulated PSED per cycle and 1-sigma stress at the utopia points for each objective function, respectively; $\Delta W_{p}^{N}$ and ${\sigma_{1\mathrm{sigma}}}^{N}$ are those at the nadir points. These values were used in the normalization of the two objective functions [25]. Table 4 shows the design variables of the initial and optimal models. The output variables of the initial and optimal models are presented in Table 5. Table 6 and Table 7 show the deformation of the BLR test board and the location of the maximum value depending on the loads of the initial and optimal models. The optimal model exhibited much less deformation of the BLR test board under thermal cycling and random vibration than the initial model. As a result, the accumulated PSED per cycle and 1-sigma stress decreased. Additionally, the locations of vulnerable solder joints were different in the initial and optimal models.

### 3.3. Calculation of the Fatigue Life of the Solder Joint

**Figure 12:**
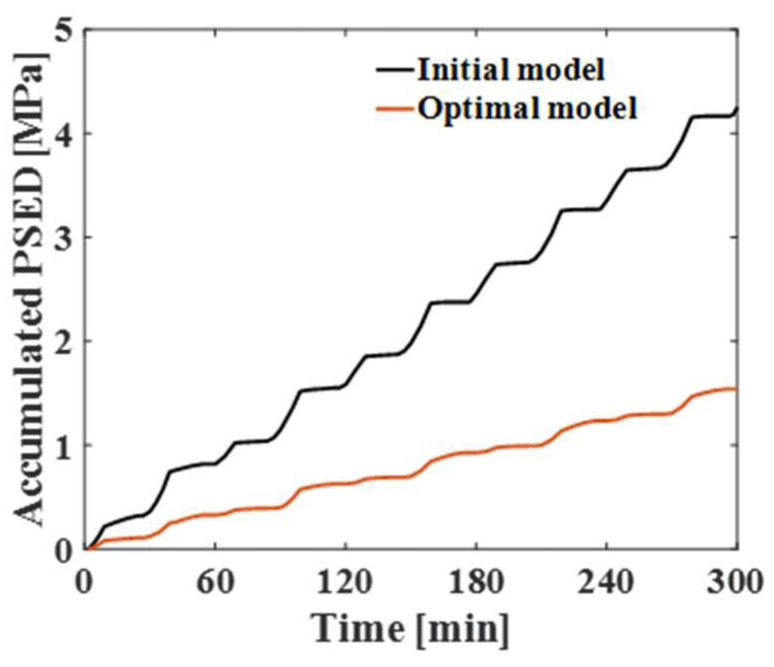
Comparison of the accumulated PSED between initial and optimal models.

**Figure 13:**
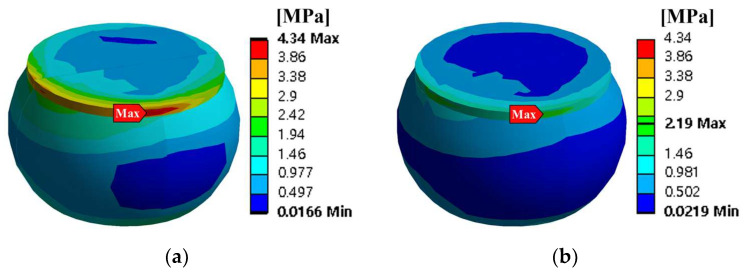
1-sigma stress contour plots of vulnerable solder ball: (**a**) initial model; (**b**) optimal model.

To calculate the fatigue life of the solder joint, the detailed structure of the solder joint shown in Figure 2b was applied to the vulnerable solder joint locations shown in Table 6 and Table 7, and thermal cycling and random vibration analyses were performed. Figure 12 presents the results of the thermal cycling analysis, which show the accumulated PSED for the vulnerable solder joint in the initial and optimal models. The values of the accumulated PSED per cycle for the initial and optimal models were 0.90 MPa and 0.31 MPa, respectively. The accumulated PSED of the optimal model was 66% lower than that of the initial model. Figure 13 shows the results of random vibration analysis, showing the contour plot of the 1-sigma stress for the vulnerable solder joint in the initial and optimal models. The maximum stress values of the initial and optimal models were 4.36 MPa and 2.14 MPa at the neck of the solder joint, respectively. The 1-sigma stress of the optimal model was about 51% lower than that of the initial model. The fatigue life of the solder joint under thermal cycling was calculated using the Morrow energy-based fatigue model with Equation (1). The fatigue exponent and fatigue ductility coefficient for SAC305 were 0.3906 and 4.504, respectively, which were obtained from Mustafa et al. [26]. We used the Steinberg three-band equation in Equation (3) to calculate the fatigue life of the solder joint under random vibration. The fatigue strength coefficient and fatigue strength exponent for SAC305 were 64.8 MPa and −0.1443, respectively, which were obtained from Yu et al. [27]. After the fatigue life of the solder joint of the optimal model was compared with that of the initial model, the fatigue life of the optimal model was found to be approximately 16 times higher under thermal cycling and 83 times higher under random vibration.

## 4. Conclusions

This study investigated the influence of screw hole position on a BLR test board to improve the reliability of BGA solder joints under thermal cycling and random vibration. The correlation between the screw hole position and the main parameters of the fatigue life was analyzed using the finite element model and sensitivity analysis of the BLR test board. Through multi-objective optimization, the optimal screw hole position that maximizes the fatigue life of solder joints was determined. The conclusions of this research are as follows:

- When both the *x_hole_* and *y_hole_* design variables of the BLR test board increase, the accumulated PSED per cycle decreases, and there is a nonlinear relationship between the design variables and the accumulated PSED per cycle. As *x_hole_* increases, the first natural frequency increases and the 1-sigma stress decreases. The increase in the natural frequency results from a higher board stiffness, which consequently decreases the 1-sigma stress.
- By performing multi-objective optimization, the optimal screw hole position that maximizes the fatigue life of the solder joint was proposed. The deformation of the BLR test board caused by thermal and vibration loading was much reduced in the optimal model compared to the initial model. As a result, the accumulated PSED per cycle decreased by 66% and the 1-sigma stress decreased by 51% in the optimal model compared to the initial model.
- The fatigue life of solder joints significantly increased in the optimal model relative to the initial model under thermal cycling and random vibration. The screw hole position was confirmed to have a significant impact on the fatigue life of solder joints. This research will contribute to improving the reliability of solder joints under thermal cycling and random vibration.

## Figures and Tables

**Table 1:** Material properties of the BLR test board.

Component	Material	Density (kg/m^3^)	Young’s Modulus (MPa)	Poisson’s Ratio (-)	CTE (ppm/°C)
PCB [3,17]	FR4	2752	26,000	0.40	18
Copper pad [3,17]	Copper	8960	117,000	0.34	17
Package [4,18]	Mold compound	2000	24,000	0.30	15
Solder mask [3,19]	Epoxy	1150	5000	0.30	30
Solder ball [3,20]	SAC305	7094	44,113.2	0.36	21

**Table 2:** Anand model constants for SAC305 [21].

Parameter	Description	Value
s	Initial value of deformation resistance (MPa)	21.00
Q/R	Activation energy/Universal gas constant (1/K)	9320
A	Pre-exponential factor (1/s)	3501
ξ	Multiplier of stress (-)	4.0
m	Strain rate sensitivity of stress (-)	0.25
$h_{0}$	Hardening constant (MPa)	180,000
$\hat{s}$	Coefficient for deformation resistance saturation (MPa)	30.2
n	Strain rate sensitivity of saturation value (-)	0.01
a	Strain rate sensitivity of hardening (-)	1.78

**Table 3:** Lower and upper limits of the design variables.

Parameter	Symbol	Lower Boundary	Upper Boundary
x-coordinate position of the screw hole (mm)	*x_hole_*	3	27
y-coordinate position of the screw hole (mm)	*y_hole_*	3	28.5

**Table 4:** Initial and optimal values of the design variables.

Parameter	Initial Model	Optimal Model
x-coordinate position of the screw hole (mm)	13.5	26.1
y-coordinate position of the screw hole (mm)	3.0	28.0

**Table 5:** Simulated output values of the initial and optimal models.

Parameter	Initial Model	Optimal Model
Accumulated PSED per cycle (MPa)	0.65	0.22
1st natural frequency (Hz)	377	623
1-sigma stress (MPa)	3.78	1.94

**Table 6:** Thermal cycling simulation results.

Response	Initial Model	Optimal Model
Total deformation of the BLR test board	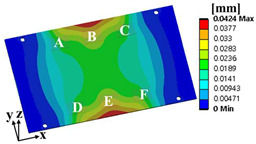	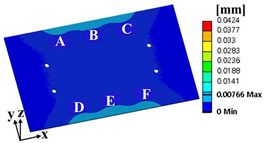
Location of the maximum accumulated PSED	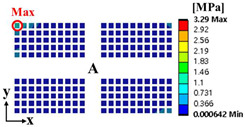	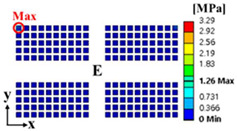

**Table 7:** Random vibration simulation results.

Response	Initial Model	Optimal Model
Directional deformation in z-axis of the BLR test board	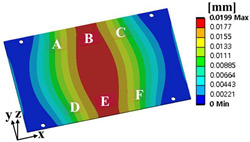	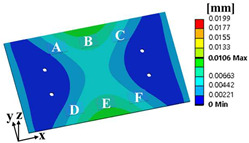
Location of the maximum 1-sigma stress	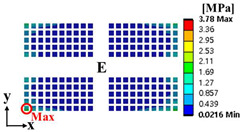	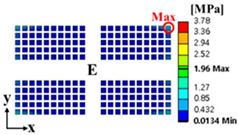

## Data Availability

Data are contained within the article.
